# Telerehabilitation Combined With Outpatient Care Reducing Rehospitalization After Acute Exacerbation of Chronic Obstructive Pulmonary Disease (COPD): A Case Report

**DOI:** 10.7759/cureus.91178

**Published:** 2025-08-28

**Authors:** Kazuaki Suyama, Shusuke Toyama, Yasuro Nawata, Kazuya Shingai, Shuta Inutsuka, Shuhei Hashimoto, Yuki Kikuchi, Hiroyuki Yoshimine, Ryo Kozu

**Affiliations:** 1 Faculty of Rehabilitation, Reiwa Health Sciences University, Fukuoka, JPN; 2 Department of Physical Therapy Science, Nagasaki University Graduate School of Biomedical Sciences, Nagasaki, JPN; 3 Department of Rehabilitation Medicine, Tagami Hospital, Nagasaki, JPN; 4 Department of Pulmonary Medicine, Tagami Hospital, Nagasaki, JPN; 5 Pulmonary Rehabilitation Center, Kirigaoka Tsuda Hospital, Kitakyushu, JPN; 6 Department of Rehabilitation Medicine, Inoue Hospital, Nagasaki, JPN; 7 Department of Rehabilitation Medicine, Dejima Hospital, Nagasaki, JPN; 8 Department of Respiratory Medicine, Inoue Hospital, Nagasaki, JPN

**Keywords:** acute axacerbation, chronic obstructive pulmonary disease, pulmonary rehabilitation, rehospitalization, telerehabilitation

## Abstract

Telerehabilitation is an emerging complement to conventional pulmonary rehabilitation for patients with chronic obstructive pulmonary disease (COPD), but its role following acute exacerbations is not well established. We report the case of a 72-year-old man with Global Initiative for Chronic Obstructive Lung Disease stage IV COPD on long-term oxygen therapy who was hospitalized for an acute exacerbation. After discharge, he initiated weekly outpatient rehabilitation. However, his physical activity declined, and he was readmitted after eight weeks. Following inpatient stabilization, he underwent an eight-week combined telerehabilitation and outpatient program. The patient showed improved adherence, with no further exacerbations. His six-minute walk distance increased by 32 m, COPD Assessment Test score improved by seven points, and the symptom domain of the St. George’s Respiratory Questionnaire improved by 10 points. This case highlights the potential of integrating telerehabilitation into outpatient care to improve continuity and prevent rehospitalization in patients with COPD.

## Introduction

Chronic obstructive pulmonary disease (COPD) is the fourth leading cause of death and represents a significant global health burden [1]. Acute exacerbation of COPD (AECOPD) contributes substantially to mortality, with hospitalization rates increasing with disease progression [2,3]. Pulmonary rehabilitation, which incorporates exercise and education, effectively reduces dyspnea, enhances exercise capacity, and lowers hospitalization rates, particularly if initiated within three months postdischarge [4]. However, limited access to rehabilitation underscores the need for innovative solutions [5].

Telerehabilitation, a remote rehabilitation approach via telecare, has demonstrated benefits comparable to conventional outpatient programs, specifically in improving exercise capacity and health-related quality of life, and in reducing hospital readmissions among patients with COPD [6,7]. Outpatient rehabilitation offers supervised, structured care that can be highly effective but may pose barriers such as transportation difficulties, scheduling conflicts, and physical burden on patients, particularly those with advanced disease [8]. In contrast, telerehabilitation improves accessibility by delivering care at home, but its challenges include limitations in hands-on assessment, variability in adherence, and the need for technical literacy and support [9]. These issues are especially pronounced in severe cases characterized by frequent AECOPD, where robust evidence for remote rehabilitation remains limited.

Integrating low-frequency outpatient rehabilitation with telerehabilitation may leverage the advantages of both approaches-ensuring structured oversight while enhancing flexibility and continuity. This combined model has the potential to support adherence, promote safety, and sustain clinical benefits. We present a case that illustrates the potential benefits of this combined approach in improving health outcomes and preventing rehospitalization following an acute exacerbation.

## Case presentation

**Figure 1:**
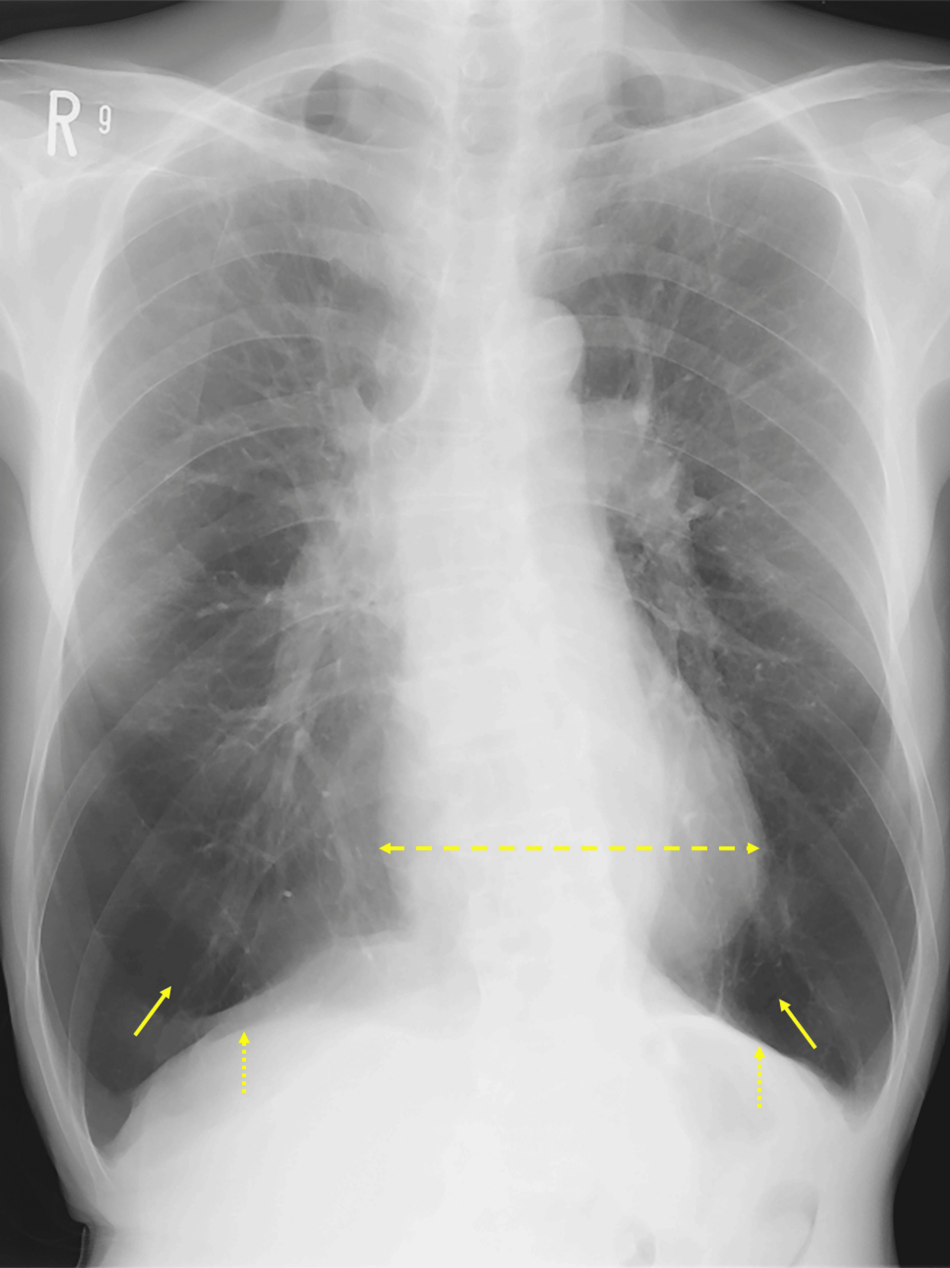
Chest radiograph showing characteristic features of advanced chronic obstructive pulmonary disease (COPD) in the present case Solid single-headed arrows indicate lung hyperinflation; dotted single-headed arrows indicate flattening of the diaphragmatic domes; dashed double-headed arrows indicate a reduced cardiothoracic ratio.

A 72-year-old man was admitted to the hospital with his second lifetime episode of AECOPD, which was diagnosed following a thorough evaluation after he presented to the emergency department with worsening dyspnea at rest. He had severe COPD (Global Initiative for Chronic Obstructive Lung Disease (GOLD) stage IV; modified Medical Research Council (mMRC) dyspnea grade 3) and had been receiving long-term oxygen therapy. Pulmonary function tests conducted prior to admission revealed a vital capacity (VC) of 1.79 L (57.4% predicted), a forced vital capacity (FVC) of 1.78 L (57.1% predicted), a forced expiratory volume in one second (FEV₁) of 0.60 L (28.8% predicted), and an FEV₁/FVC ratio of 33.7%, consistent with GOLD stage IV airflow limitation. Prior to admission, he had led a largely sedentary lifestyle, attending weekly outpatient visits by car, which he drove himself, but otherwise remained indoors with limited physical and social engagement. At the time of hospitalization, physical examination revealed a barrel-shaped chest, diminished breath sounds, Hoover’s sign, and sternocleidomastoid muscle hypertrophy. Chest radiography revealed hyperinflated lungs with flattened diaphragmatic domes and a decreased heart size (Figure 1). Arterial blood gas under 2.0 L/minute nasal oxygen revealed compensated respiratory acidosis (pH 7.31, PaCO₂ 59.6 mmHg, PaO₂ 59 mmHg, HCO₃^-^ 29.5 mmol/L). Despite being underweight (body mass index, 15.6 kg/m²; Geriatric Nutritional Risk Index, 86.1), laboratory findings showed no significant inflammation, anemia, or renal dysfunction (Table 1).

**Table 1:** Laboratory findings at admission and reference ranges Reference ranges are derived from gender- and age-specific intervals for Japanese individuals aged 65-80 years, as reported by Yamakado et al. [10], and for creatinine, the original values in µmol/L were converted to mg/dL by dividing by 88.4 to ensure consistency in units.

Parameter	Result	Unit	Reference range (male, 65-80 years)
C-reactive protein (CRP)	0.03	mg/L	<0.3
Hemoglobin (Hb)	133	g/L	133-163
Hematocrit (Ht)	39.4	%	39.4-47.9
Red blood cell count (RBC)	4.15	×10¹²/L	4.15-5.18
Creatinine (Cre)	0.64	mg/dL	0.64-1.13
Estimated GFR (eGFR)	93.4	mL/min/1.73 m²	50-93
Albumin (Alb)	41.2	g/L	38.7-47.3
Total protein (TP)	63.1	g/L	64.3-78.4

He was admitted and received comprehensive multidisciplinary inpatient treatment in accordance with the GOLD guidelines [11], including systemic corticosteroids, bronchodilators, prophylactic antibiotics, and oxygen therapy titrated based on arterial blood gas results. Pulmonary rehabilitation was administered twice daily, five to six days/week, and included breathing exercises, aerobic and resistance training, and activities to improve functional capacity. Nutritional and pharmaceutical support were provided. Education and psychological counseling were included to promote long-term disease management.

Phase 1: only-outpatient rehabilitation program

The outpatient rehabilitation program was conducted face-to-face and included conditioning exercises such as breathing exercises, manual breathing assist, and stretching of accessory respiratory muscles. It also comprised resistance training, ergometer exercises, and physical activity counseling. The ergometer exercise intensity was set at 40% of the predicted maximum workload, derived from the six-minute walk distance (6MWD) [12], with each session lasting 20 minutes and tailored to achieve a modified-Borg Scale (mBS) [13] rating of four to five. Dietary intake and bronchodilator usage were self-reported by the patient without direct supervision.

After discharge, the patient began weekly outpatient rehabilitation. Over the following weeks, his symptoms gradually worsened, with increasing dyspnea and reduced exercise tolerance, ultimately leading to hospital readmission. During low-intensity physical activity at a level of two to three metabolic equivalents of task, the patient reported an mBS score exceeding five, which contributed to decreased participation in both supervised and unsupervised sessions. At week 8, the patient developed wheezing and dyspnea, prompting readmission. This episode met the diagnostic criteria for AECOPD as defined by the GOLD guidelines [11]. Quantitative assessments revealed a 70-m decrease in the 6MWD, a three-point increase in the COPD Assessment Test (CAT; © GSK group of companies) [14] score, and a seven-point worsening in the symptom domain of the St. George’s Respiratory Questionnaire (SGRQ) [15]. The Client Satisfaction Questionnaire-8 (CSQ-8) [16] score for this program was 21 points. Notably, lower ratings were observed for the items assessing whether the patient received the kind of treatment he wanted, and whether he received sufficient time and support to address his problems.

Telerehabilitation systems

**Figure 2:**
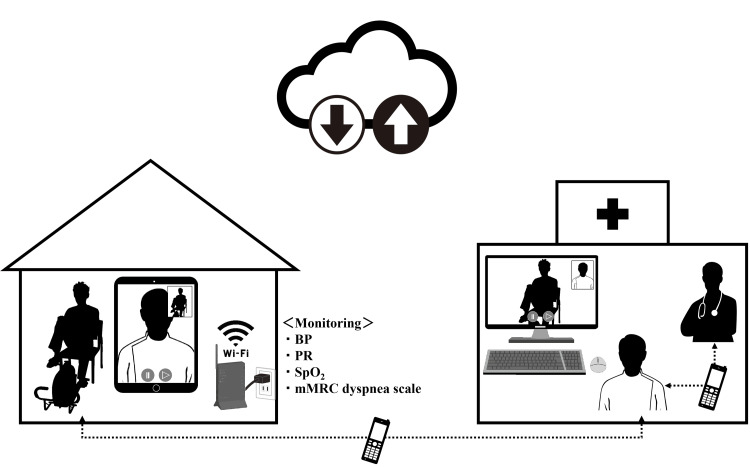
Telerehabilitation system for patients with chronic obstructive pulmonary disease (COPD) The schematic was created by combining royalty-free elements obtained from AC Illust (ACworks Co., Ltd., Osaka, Japan) (https://www.ac-illust.com/) with original illustrations and photographs produced entirely by the authors. BP, blood pressure; COPD, chronic obstructive pulmonary disease; PR, pulse rate; SpO_2_, percutaneous oxygen saturation

Following inpatient stabilization, we developed a telerehabilitation system specifically designed for patients with COPD (Figure 2). The system’s core is a host terminal PC at the hospital, equipped with the YaDoc® online medical care system (Integrity Healthcare Co., Ltd., Tokyo, Japan). Additionally, we provided patients with a tablet computer (iPad®, Apple Inc., Cupertino, CA) to facilitate a reliable internet communication environment for video conferencing. For continuous monitoring and effective telerehabilitation implementation, patients were provided with devices for real-time monitoring of physiological parameters, including a blood pressure monitor, pulse oximeter, and a variable-load portable cycle ergometer (Terasu Ergo®, Showa Denki Co., Ltd., Osaka, Japan) as exercise training equipment. This setup enabled real-time health monitoring during exercise sessions via video. A comprehensive management system also allowed direct communication between the patient’s home and medical staff or attending physicians in the event of any adverse events.

The telerehabilitation system was conducted in accordance with the ethical principles of the Declaration of Helsinki and complied with the ethical guidelines for medical research involving human subjects. The cases’ personal information was protected to ensure anonymity, and written informed consent was obtained after providing sufficient verbal and written explanations. The system was also approved by the Human Ethics Review Committee of Reiwa Health Sciences University (approval number: 24-005), and a confidentiality agreement was signed by medical institutions with Integrity Healthcare Inc. The system was implemented after thorough pilot testing to ensure safety and reliability.

Using this system, a telerehabilitation program was implemented via online videoconferencing and mirrored the outpatient rehabilitation in structure and content. They encompassed conditioning (respiratory gymnastics), resistance training, ergometer exercises (following the same protocol as in outpatient rehabilitation), and physical activity counseling. In addition, dietary intake and bronchodilator usage were visually confirmed through the video interface during the sessions. Patient education was also provided as needed, enhancing the program's adaptability and responsiveness to the patient’s condition.

Phase 2: combined outpatient and telerehabilitation program

After stabilization, Phase 2 began, consisting of a combined outpatient and telerehabilitation program. Over the eight-week period, the patient experienced no episodes meeting the diagnostic criteria for AECOPD. He gradually increased his physical activity at home, consistently attended supervised sessions, and progressively engaged in unsupervised training. Quantitative measures at the end of the eight-week period showed a 32-m gain in the 6MWD, a seven-point reduction in the CAT score, and a 10-point enhancement in the symptom domain of the SGRQ. The CSQ-8 score increased from 21 to 26 points, with higher ratings recorded for the items assessing whether the patient received the kind of treatment he wanted, and whether he received sufficient time and support to address his problems.

**Figure 3:**
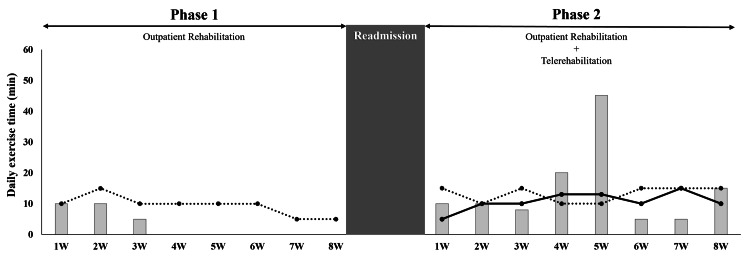
Changes in supervised and unsupervised exercise time during each phase The dotted line represents the supervised exercise time during outpatient rehabilitation, the solid line represents the supervised exercise time during telerehabilitation, and the bar graph indicates the unsupervised exercise time at home.

The rehabilitation progress and outcomes for both phases are summarized in Figure 3 and Table 2, which allow for a direct comparison between the outpatient-only and the combined rehabilitation programs.

**Table 2:** Changes in outcomes for each session All assessment tools and scales are either in the public domain or used in accordance with their respective licensing requirements. The CSQ-8 was used under a formal license agreement (documentation available). The SGRQ was used with permission from St George’s, University of London (permission letter available). The COPD Assessment Test was used with the required copyright notice: ©GSK group of companies. AECOPD, acute exacerbation of COPD; CAT, COPD Assessment Test; CSQ-8, Client Satisfaction Questionnaire-8; GNRI, Geriatric Nutritional Risk Index; SGRQ, St. George’s Respiratory Questionnaire; 6MWD, six-minute walk distance

	Phase 1			Phase 2		
	Baseline	8W	Δ	Baseline	8W	Δ
Body weight, kg	40	38.2	-1.8	42.7	43.2	0.5
GNRI	88	86	-2	91	91.4	0.4
6MWD, m	290	220	-70	244	276	32
CAT, score	16	19	3	23	16	-7
SGRQ total	59	84	25	55	56	1
Symptoms	88	95	7	77	67	-10
Activity	80	93	13	80	80	0
Impact	38	75	37	34	39	5
CSQ-8, score	-	21	-	-	26	-
Diagnosed AECOPD	-	Yes	-	-	No	-

## Discussion

We described a patient with AECOPD who showed improved exercise capacity and health-related quality of life, along with no further exacerbations, following a combined outpatient and telerehabilitation program. These improvements occurred after a period of clinical decline during outpatient rehabilitation alone, which ultimately led to hospital readmission. The improvement in functional capacity, as indicated by the 6MWD, and reductions in symptom burden, as measured by the CAT and SGRQ scores, may reflect the synergistic benefits of combining supervised in-person rehabilitation with home-based support through telerehabilitation. Indeed, despite the shorter duration and lower frequency compared to previous studies [17], the observed changes in the 6MWD and CAT exceeded the minimal clinically important differences [18,19], suggesting meaningful clinical improvement. Hansen et al. reported that a 10-week telerehabilitation program conducted three times per week in patients with GOLD stage III-IV COPD resulted in a 17.2 m increase in 6MWD and a 1.7-point decrease in CAT scores, outcomes comparable to conventional outpatient rehabilitation [17]. These findings suggest that combining outpatient rehabilitation and telerehabilitation yields synergistic effects that enhance exercise training outcomes in this case.

During Phase 1, the patient’s SGRQ total and subdomain scores worsened considerably. We believe this was primarily due to clinical deterioration, including increased dyspnea, reduced exercise tolerance, and subsequent rehospitalization. Notably, the SGRQ was administered just prior to readmission, during which time the patient was experiencing marked physical and psychological distress. Since the SGRQ is a self-reported, symptom-based measure, the scores likely reflected the patient’s acute perception of worsening health status at that specific time point. Similarly, the CSQ-8 score was lower during Phase 1, particularly in items assessing whether the patient received the kind of service and amount of help he wanted. This may suggest a mismatch between the patient’s expectations and the care provided. Because rehabilitation was limited to once-weekly outpatient sessions with little support outside these encounters, the patient may have perceived this as insufficient. Additionally, the worsening of symptoms and rehospitalization during this period likely influenced his overall satisfaction.

The integration of telerehabilitation in Phase 2 allowed for care to be adapted to the patient’s home environment, potentially increasing engagement in self-directed exercise and improving symptom management. Telemonitoring and feedback provided through video conferencing may enhance patients’ health awareness and adherence, supporting behavioral changes and self-management. This observation aligns with previous reports suggesting that telemonitoring, even when not accompanied by exercise training, combined with individualized coaching, can help maintain patient motivation and prevent rehospitalization [20].

Although these findings are encouraging, this study has several limitations that should be acknowledged. This report describes a single case, and the outcomes may not be generalizable. Moreover, there were baseline differences between the outpatient-only and the combined rehabilitation phases, including disease severity and timing relative to hospitalization, which may limit the comparability of the outcomes between phases. In addition, the benefits were limited to a relatively short follow-up period. While no adverse events or dropouts occurred, formal assessments of exercise adherence and system usability were not conducted. Furthermore, psychological aspects such as anxiety and depression were not fully evaluated, and therefore conclusions regarding mental health outcomes cannot be drawn from this case. Nonetheless, the patient's consistent participation, lack of complications, and favorable clinical course suggest practical feasibility. Further studies with larger cohorts and longer observation periods are required to validate these findings and to clarify the real-world challenges of implementing telerehabilitation in diverse settings.

This case highlights the potential value of integrating telerehabilitation with standard rehabilitation for managing AECOPD. This approach may provide a practical strategy to support continuity of care, promote physical activity, and reduce the risk of rehospitalization, particularly in patients who face barriers to traditional outpatient programs.

## Conclusions

This case demonstrates that integrating telerehabilitation with conventional outpatient programs can help maintain continuity of care and improve clinical outcomes for patients recovering from AECOPD. The patient demonstrated improvements in exercise capacity and health-related quality of life with no further exacerbations during the intervention period. These findings indicate that telerehabilitation may help overcome logistical barriers to traditional outpatient programs and provide meaningful clinical benefits when integrated into multidisciplinary care. Further investigations are warranted to explore the broader applicability of this approach in similar patient populations.
